# Case report of intracranial neuroendocrine carcinoma

**DOI:** 10.3389/fonc.2026.1776690

**Published:** 2026-04-16

**Authors:** Lisha Ren, Qianyong He, Weili Wu, Jinhua Long, Yuanyuan Li, Xiaoxiao Chen, Xiuyun Gong, Feng Jin, Xiuling Luo

**Affiliations:** 1 Department of Oncology, Affiliated Hospital of Guizhou Medical University, Guiyang, Guizhou, China; 2 Department of Head and Neck Oncology, Affiliated Tumor Hospital of Guizhou Medical University, Guiyang, Guizhou, China; 3 School of Clinical Medicine, Guizhou Medical University, Guiyang, Guizhou, China

**Keywords:** case report, diagnosis, intracranial neuroendocrine carcinoma, outcome, treatment

## Abstract

Intracranial neuroendocrine carcinoma (NEC) is a highly uncommon malignancy, accounting for approximately 0.74% of cases. It is characterized by rapid infiltration and poor survival rates. This case report details a 51-year-old woman who presented with headaches. Magnetic resonance imaging (MRI) identified an enhancing lesion in the right temporal lobe, and the diagnosis was confirmed by immunohistochemical (IHC) analysis. The patient underwent surgical resection, followed by chemoradiotherapy for recurrence, and subsequent Gamma Knife radiosurgery combined with bevacizumab. Notably, she has achieved a postoperative survival exceeding four years to date. This report comprehensively describes the clinical presentation, diagnostic workup, multidisciplinary treatment course, and favorable outcome, highlighting the potential for prolonged survival with aggressive, multimodal management.

## 1 Introduction

Neuroendocrine tumors (NETs) originate from the diffuse neuroendocrine system, with fewer than 0.74% arising intracranially. This rarity complicates preoperative diagnosis and contributes to a generally poor prognosis (1). Although these tumors are most frequently located in the digestive system (2), they can, on rare occasions, develop within the brain. Cheng et al. (3) have compiled data from nine documented cases of primary intracranial origin to date. This report presents a new case of intracranial neuroendocrine carcinoma and analyzes it alongside the existing literature to improve the understanding and clinical management of this disease.

## 2 Case report

**Figure 1:**
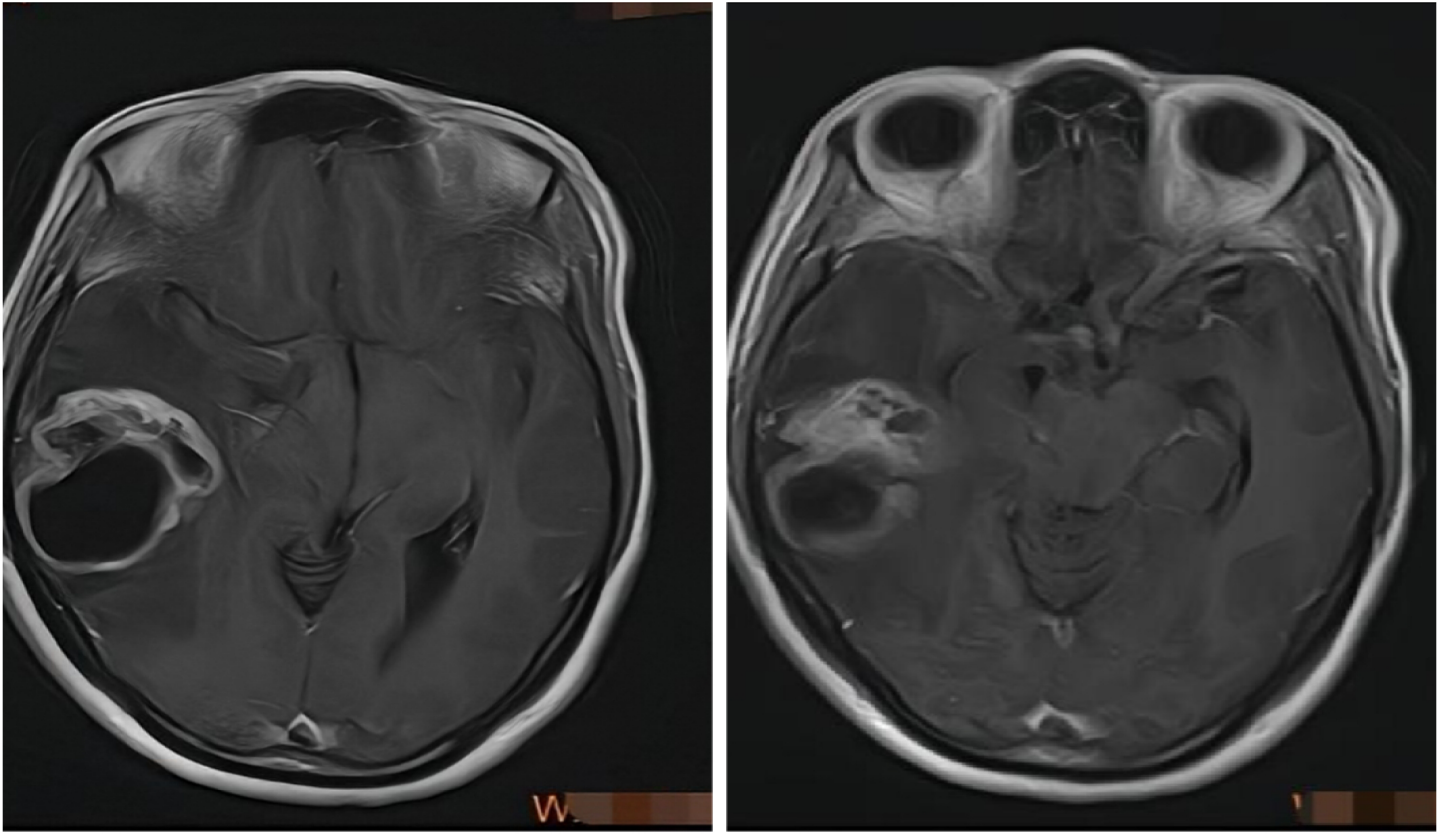
T1-weighted sagittal MRI showed an enhancing lesion in the right temporal lobe with enhancing signals.

A 51-year-old woman was admitted to our department with a four-month history of headaches. Biochemical and endocrinological tests—including thyroid function, adrenocortical function, and female hormone panels—were all within normal limits. Magnetic resonance imaging (MRI) revealed a prominently enhancing lesion in the right temporal lobe, showing hyperintensity on T1-weighted images (**Figure 1**). The preoperative diagnosis was suspected high-grade glioma. Preoperative abdominal and chest computed tomography (CT) scans showed no abnormalities.

**Figure 2:**
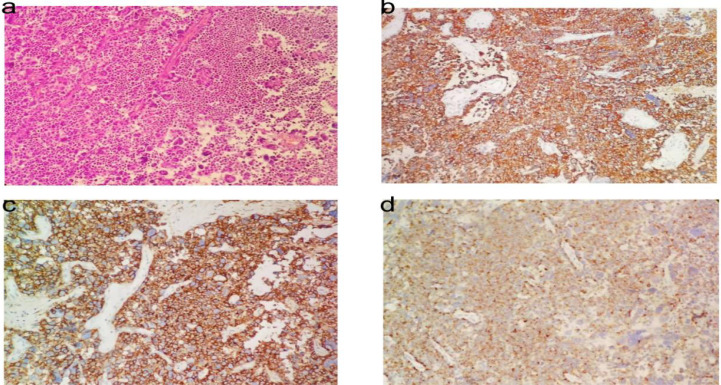
The pathological results of sheets of cells with large cells with prominent nuclei (H&E, 200x) **(A)**. Tumor cells were immunohistochemical positive for Syn **(B)**, CD56 **(D)**, and CgA **(D)**, (IHC 200×).

**Figure 3:**
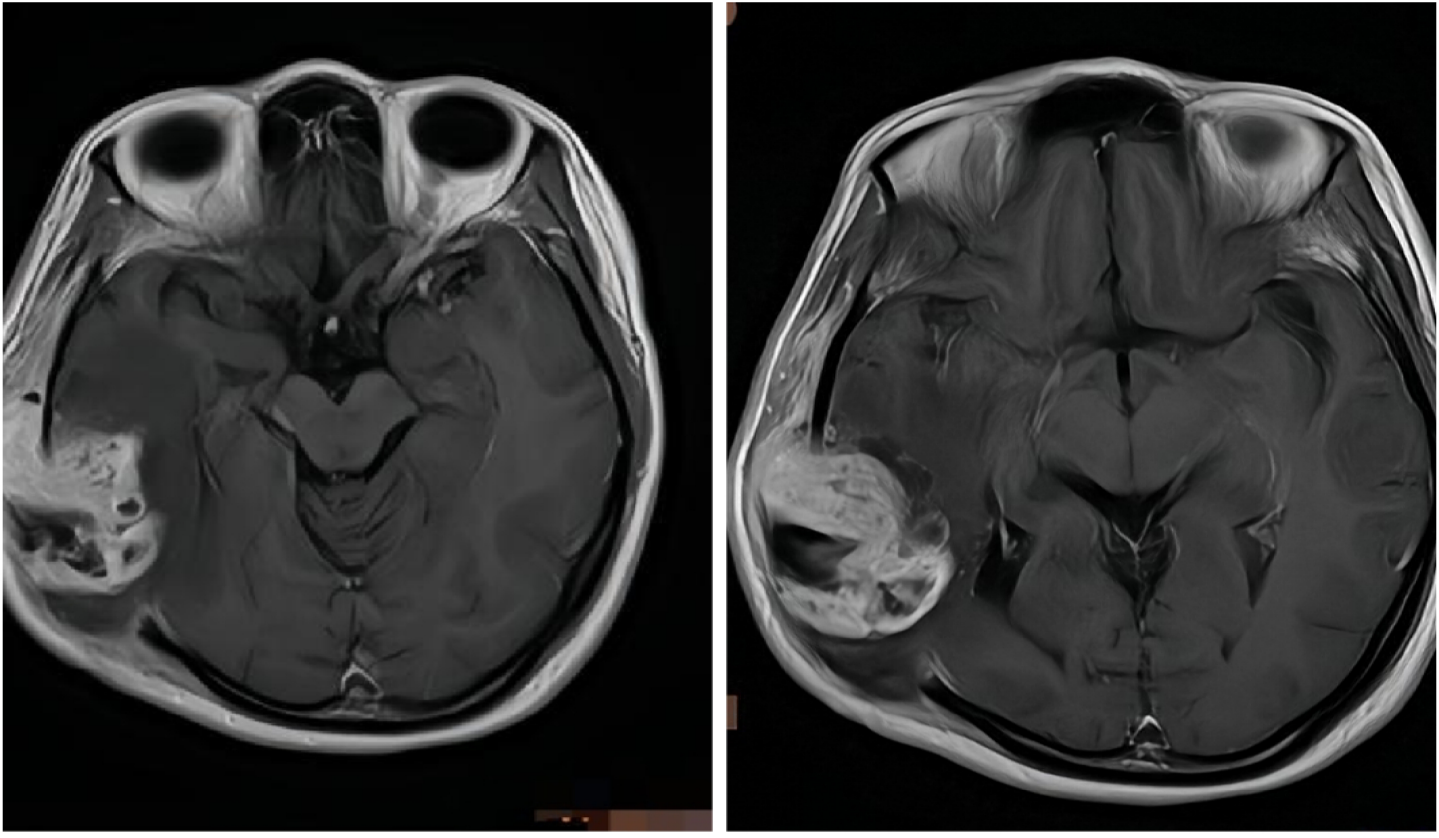
After surgery one month later, T1-weighted sagittal MRI showed that the solid component of the right temporal soft tissue mass was more enlarged than before.

The patient subsequently underwent tumor resection via a right temporal craniotomy. Morphological analysis showed that the tumor was composed of variably sized cells with marked atypia, arranged in dispersed and sheet-like patterns, indicative of active proliferation (**Figure 2A**). The tumor cells tested positive for neuroendocrine markers, including synaptophysin (Syn) (**Figure 2B**), and for epithelial markers such as CD56 and chromogranin A (CgA) (**Figures 2C, D**). Pan-cytokeratin (CK) immunostaining was positive, while CD20, S-100, HMB45, and CK20 were negative. The Ki-67 proliferation index was 70%. Based on these morphological and immunohistochemical findings, and according to the WHO 2022 Classification of Endocrine and Neuroendocrine Tumors (4), the final diagnosis was extrapulmonary high-grade large-cell neuroendocrine carcinoma, suggesting possible intracranial primary origin, but clinical exclusion of metastasis is required. At a follow-up visit over one month post-surgery, cranial MRI indicated enlargement of the solid component of the right temporal soft-tissue mass and increased surrounding edema compared to prior imaging (**Figure 3**). Chemoradiotherapy was recommended to control disease progression.

The patient received six cycles of chemotherapy with cisplatin and temozolomide (body surface area: 1.40 m²; temozolomide: 150 mg/m²/day, days 1–5; cisplatin: 80 mg/m², days 1–2). The choice of this regimen was based on two points: first, temozolomide has good blood-brain barrier permeability and potential activity against various central nervous system tumors, including NEC; second, existing studies suggest that temozolomide shows certain efficacy in progressive high-grade NEC (5). Adverse effects included grade 3 leukopenia and neutropenia, as well as grade 1 elevated creatinine and vomiting, all of which improved with supportive care. Additionally, radiotherapy was delivered using the volumetric modulated arc therapy (VMAT) technique to the postoperative residual lesion and tumor bed, with a total dose of 60.06 Gy in 30 fractions. Similar consolidation radiotherapy approaches have been employed in head and neck neuroendocrine carcinomas to improve local control (6). No significant radiotherapy-related side effects were observed.

**Figure 4:**
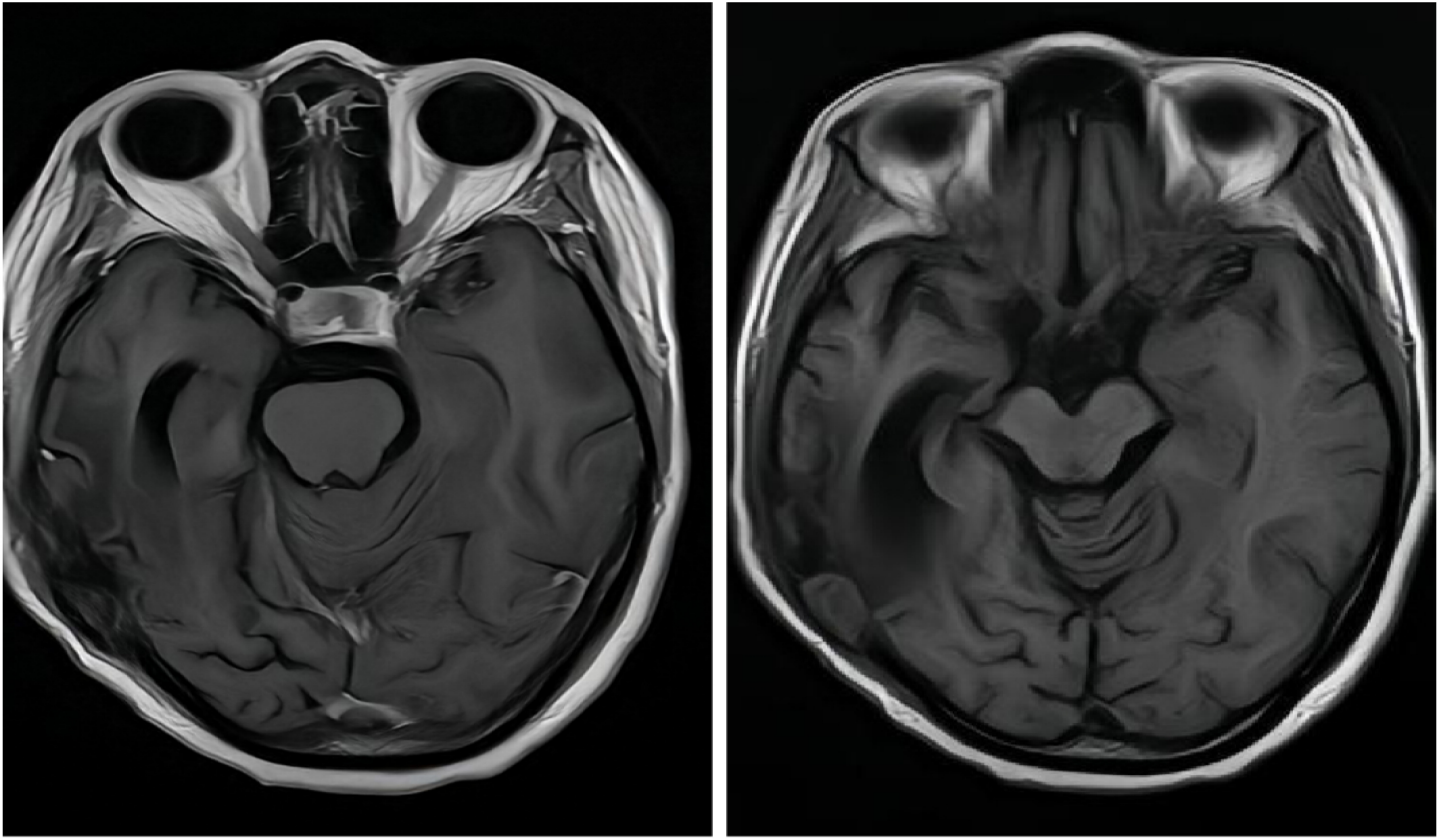
After Six cycles of chemotherapy and radiotherapy, completion of treatment confirmed complete disease remission through T1-weighted sagittal MRI.

Post-treatment cranial MRI confirmed complete disease remission (**Figure 4**), and the patient was placed on a schedule for imaging follow-up every three months.

**Figure 5:**
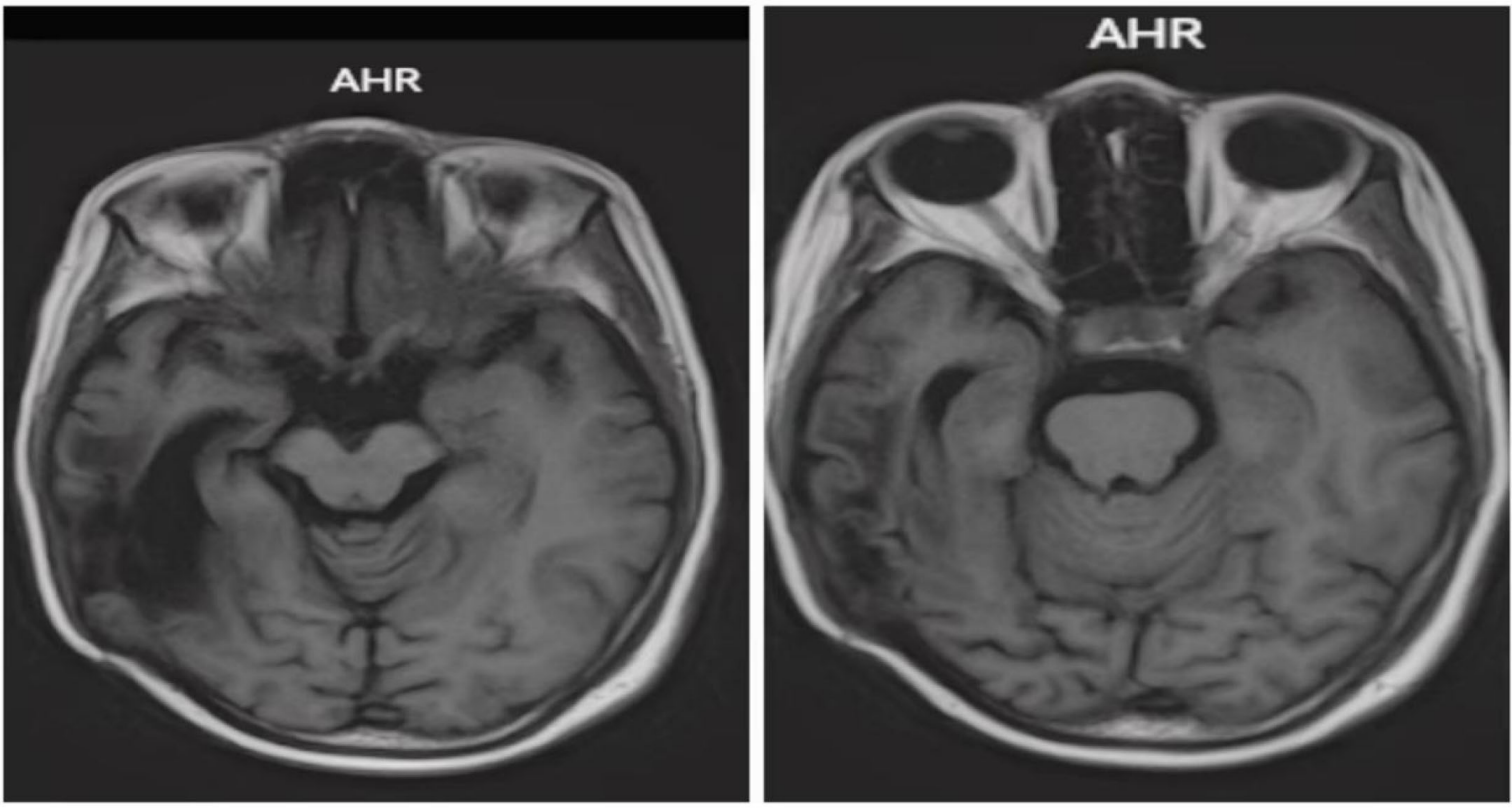
Three years postoperatively, revealed an enlarged, irregularly thickened, and heterogeneously enhancing cystic lesion at the surgical margin.

Three years after the initial surgery, imaging studies demonstrated disease progression, showing an enlarged, irregularly thickened, and heterogeneously enhancing cystic lesion at the surgical margin (**Figure 5**), indicating local recurrence. The patient then underwent two sessions of Gamma Knife radiosurgery as a local boost. First plan:A total of 4 target volumes were designed for irradiation, with the following target volume doses:

1. 15 Gy, 50% isodose line (Max 30 Gy); tumor volume: 1.96 cm³, number of target points: 24.
2. 12.3 Gy, 50% isodose line (Max 24.6 Gy); tumor volume: 2.17 cm³, number of target points: 4.
3. 12.3 Gy, 50% isodose line (Max 24.6 Gy); tumor volume: 1.43 cm³, number of target points: 21.
4. 12.3 Gy, 50% isodose line (Max 24.6 Gy); tumor volume: 7.34 cm³, number of target points: 6.

The second plan: Two target volumes were designed.

- Target Volume 1: Dose 12.3 Gy (50% isodose line, Max 24.6 Gy), tumor volume 2.60 cm³, 5 target points.

- Target Volume 2: Dose 12.3 Gy (50% isodose line, Max 24.6 Gy), tumor volume 1.79 cm³, 6 target points.

**Figure 6:**
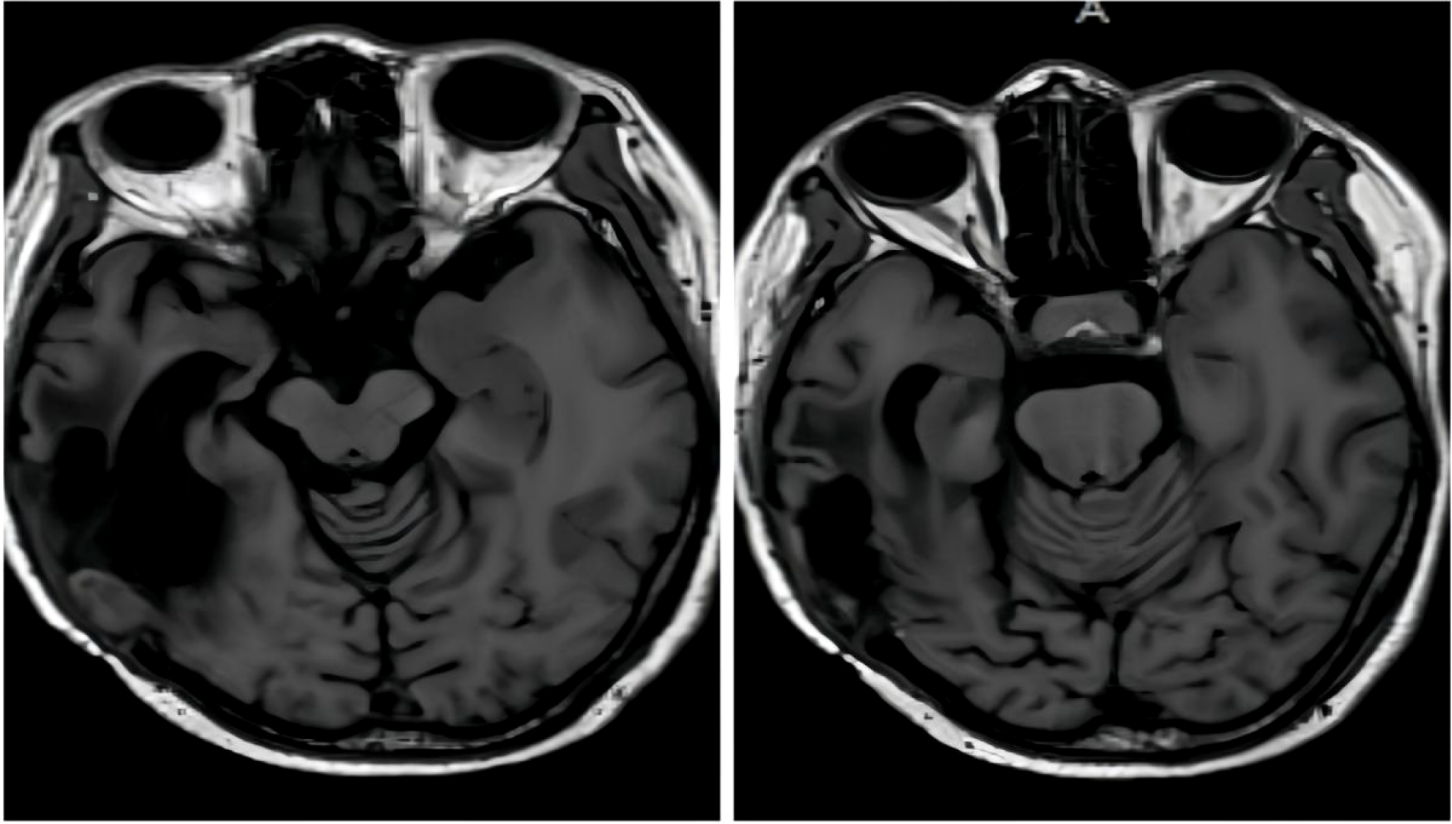
Post-treatment MRI (T1-weighted) after combined Gamma Knife and bevacizumab therapy revealed no significant abnormal signal intensity.

The target points of each lesion were reasonably distributed with satisfactory conformity, and important structures around the lesions were within the safe irradiation range. The treatment process was smooth. At the end of the operation, the patient reported no discomfort. The stereotactic frame was removed, the minimally invasive wound was dressed, and the patient was returned to the ward for intravenous therapy. During this treatment, to manage refractory peritumoral edema caused by tumor recurrence, bevacizumab was administered concurrently (every 3 weeks for a total of 4 cycles). Concurrently with bevacizumab to manage intracranial edema. Follow-up examinations every three months thereafter have shown stable disease. However, the patient developed hearing impairment and reduced muscle strength in all four limbs. As of August 2025, the patient has survived for four years after surgery, and follow-up imaging shows a disease-free status (**Figure 6**).

## 3 Discussion

This case report describes a rare instance of intracranial NEC and details the process of achieving long-term disease-free survival (>4 years) through a multimodal comprehensive treatment strategy including surgery, chemoradiotherapy, Gamma Knife, and targeted therapy. This outcome is significantly better than the reported survival of only 3 months for patients with high-grade primary brain neuroendocrine tumors in the literature (1), suggesting that aggressive comprehensive treatment may significantly improve the prognosis for some patients. However, the diagnosis and treatment process of this case also raise considerations regarding several key issues. First, the diagnostic challenge of primary intracranial NEC. It must be acknowledged that determining an “intracranial primary” NEC is a diagnosis of exclusion, with the gold standard being the failure to identify an extracranial primary site through systemic examination. In this case, we performed contrast-enhanced CT scans of the chest and abdomen, which were negative, providing preliminary evidence for the diagnosis. However, CT alone is insufficient to completely exclude small or occult extracranial primary tumors, especially NENs of the lung and digestive tract. The assessment of intracranial metastases from neuroendocrine tumors requires a high index of suspicion, and functional imaging plays a crucial role (7). The most important limitation of this case is the inability to perform more comprehensive whole-body functional imaging, such as ^68^Ga-DOTATATE PET/CT or ¹^8^F-FDG PET/CT, the latter having higher sensitivity for detecting high-grade NEC with high metabolic activity. Third, the rationale for treatment strategy selection. The cisplatin + temozolomide regimen used in this case is not the standard first-line regimen for high-grade NEC (typically cisplatin + etoposide). Our choice was based on the following considerations: 1) Blood-brain barrier permeability: As a foundational drug for central nervous system tumors, temozolomide effectively penetrates the blood-brain barrier, offering a theoretical advantage for patients with intracranial lesions and potential microscopic residual disease. 2) Literature support: The study by Welin et al. (8) showed that temozolomide-based chemotherapy regimens can still achieve disease control in some patients with high-grade NEC who have progressed after first-line chemotherapy, with a median progression-free survival of 6 months. At the recurrence stage, our combination with bevacizumab had two main purposes: first, to leverage its anti-angiogenic effect in an attempt to control tumor growth; second, to utilize its property of reducing vasogenic edema to rapidly alleviate patient symptoms, thereby creating conditions for subsequent Gamma Knife treatment. The long-term survival in this case is an encouraging observation, but we cannot attribute it to any single treatment modality. Rather, it should be attributed to the cumulative effect of the multimodal, multi-line treatment strategy of “surgery + chemoradiotherapy + Gamma Knife + bevacizumab.”

Finally, comparison with previous literature and limitations of this study. A review of the literature shows that intracranial NEC is mostly reported in case studies, with significant variability in survival. In the two cases reported by Liu et al. (1), one patient with a high-grade tumor survived only 3 months post-surgery. Tamura et al. (9) described a primary brain NET that was managed with surgical resection, but the long-term outcome remained guarded. Similarly, Rai et al. (10) and Caro-Osorio et al. (11) reported aggressive large-cell neuroendocrine carcinomas with intracranial involvement, highlighting the generally poor prognosis. The 4-year disease-free survival in this case is truly rare in the existing literature. We speculate that this might be related to the following factors: 1) The patient was sensitive to initial chemoradiotherapy, achieving complete remission; 2) At recurrence, the lesion was localized, suitable for local salvage therapy (Gamma Knife) 3) The combination with bevacizumab effectively controlled edema and possible microvascular proliferation. However, this study has significant limitations: 1) Single case observation, unable to draw generalizable conclusions; 2) Inability to definitively confirm intracranial primary origin; 3) Limited level of evidence for some treatment decisions. Therefore, the value of this report lies in providing a potentially effective treatment approach for clinicians managing such rare tumors and emphasizing the importance of MDT and individualized treatment.

## 4 Conclusion

Intracranial neuroendocrine carcinoma is a highly uncommon malignancy. Pathological examination remains the gold standard for diagnosis. Early detection and prompt intervention with multimodal therapy, including chemoradiation and targeted agents, may improve the prognosis of this disease.

## Data Availability

The raw data supporting the conclusions of this article will be made available by the authors, without undue reservation.
